# How new clinical roles in primary care impact on equitable distribution of workforce: a retrospective study

**DOI:** 10.3399/BJGP.2023.0007

**Published:** 2023-08-22

**Authors:** Joseph Hutchinson, Yiu-Shing Lau, Matt Sutton, Kath Checkland

**Affiliations:** Centre for Primary Care and Health Services Research, University of Manchester, Manchester.; Centre for Primary Care and Health Services Research, University of Manchester, Manchester.; Centre for Primary Care and Health Services Research, University of Manchester, Manchester.; Centre for Primary Care and Health Services Research, University of Manchester, Manchester.

**Keywords:** general practice, health services research, inequality primary health care, workforce

## Abstract

**Background:**

There are inequalities in the geographical distribution of the primary care workforce in England. Primary care networks (PCNs), and the associated Additional Roles Reimbursement Scheme (ARRS) funding, have stimulated employment of new healthcare roles. However, it is not clear whether this will impact inequalities.

**Aim:**

To examine whether the ARRS impacted inequality in the distribution of the primary care workforce.

**Design and setting:**

A retrospective before-and-after study of English PCNs in 2019 and 2022.

**Method:**

The study combined workforce, population, and deprivation data at network level for March 2019 and March 2022. The change was estimated between 2019 and 2022 in the slope index of inequality (SII) across deprivation of full-time equivalent (FTE) GPs (total doctors, qualified GPs, and doctors-in-training), nurses, direct patient care, administrative, ARRS and non- ARRS, and total staff per 10 000 patients.

**Results:**

A total of 1255 networks were included. Nurses and qualified GPs decreased in number while all other staff roles increased, with ARRS staff having the greatest increase. There was a pro- rich change in the SII for administrative staff (−0.482, 95% confidence interval [CI] = −0.841 to −0.122, *P*<0.01) and a pro- poor change for doctors-in-training (0.161, 95% CI = 0.049 to 0.274, *P*<0.01). Changes in distribution of all other staff types were not statistically significant.

**Conclusion:**

Between 2019 and 2022 the distribution of administrative staff became less pro-poor, and doctors-in-training became pro-poor. The changes in inequality in all other staff groups were mixed. The introduction of PCNs has not substantially changed the longstanding inequalities in the geographical distribution of the primary care workforce.

## INTRODUCTION

The inverse care law suggests that healthcare availability is inversely related to the need for that health care.^1^ This has been shown in general practice, with lower staff numbers relative to need in deprived areas. This article focused on this form of inequality.^2^^–^6 Primary care is an important contributor to cost-effective and equitable health systems.^2^^,^^7^ In the UK, such care is provided by general practices. Most practices are a partnership of primary care physicians and GPs, who work with employed staff to provide services to the NHS.

Some previous policies have attempted to address workforce inequalities, such as the Equitable Access to Primary Medical Care scheme, which sought to increase the number of general practices in deprived areas. The scheme had modest success improving availability in deprived areas, but was time-limited.^8^ More recently, inequalities have been shown to have widened, with the number of GPs falling fastest in the most deprived areas.^9^ This results in deprived populations having a higher risk of workforce supply–demand mismatch.^10^ This undersupply of GPs could possibly be compensated for by the employment of other healthcare workers, but evidence for this action is minimal and sometimes conflicting.^8^^,^^11^^–^13

An important development in English general practice is the introduction of primary care networks (PCNs). PCNs are collaborations of geographically contiguous practices covering populations of around 30 000–50 000 people, providing additional services to their combined populations.^14^ More than 50% of the funding for PCNs comes through the Additional Roles Reimbursement Scheme (ARRS).^15^ This scheme reimburses PCNs for the costs of employing additional healthcare staff, such as care coordinators. It is intended that this scheme will stimulate employment in general practice and address shortfalls in healthcare availability.

The ARRS funding is adjusted for workload differences by the Carr-Hill formula. The workload factors it considers are the age, sex, and additional needs of the network’s patients, as well as list turnover, rurality, and input prices. Resultantly, more funding is provided for network populations with relatively higher needs, such as those in deprived areas. As such, it might be expected to see increased ARRS employment in deprived areas. However, the Carr-Hill formula is criticised for underestimating the impact from deprivation.^16^ Moreover, increased ARRS employment to compensate for inequalities will be impacted by factors beyond funding. This may explain the incomplete utilisation of allocated ARRS funding.^17^ The employment of ARRS staff might also influence the employment of other staff. It is therefore unclear whether the ARRS will impact inequalities in the geographical distribution of the primary care workforce. To address this issue, the study analysed whether ARRS widened or narrowed this inequality in staff working in general practice, both through the ARRS and not.

**Table:** How this fits in

There is inequality in the geographical distribution of the primary care workforce in England. The primary care network Additional Roles Reimbursement Scheme (ARRS) was introduced to allow groups of practices to employ extra staff to relieve workload pressures. The study found the ARRS was associated with both pro-rich and pro-poor changes in the geographical distribution of staff. The only significant changes were that the distribution of administrative staff became less pro-poor (*P*<0.01), and doctors-in-training became pro-poor (*P*<0.01). It is reassuring that the ARRS has not exacerbated existing inequalities in clinical staff distribution. However, it is recommended that policymakers should consider how future manifestations of the scheme could be adjusted to tackle inequalities. Further, networks may choose to actively deploy staff to differentially support more deprived aspects of their community.

## METHOD

### Study design

A retrospective before-and-after study was conducted using data from March 2019 and March 2022. March 2019 was chosen to align with the deadline for the enrolment in the PCN contract.

### Data sources

#### Patient, demographic, and community data

A complete list of PCNs was gathered from NHS Digital’s Organisational Data Service (ODS) in August 2022, which maintains a list of NHS providers.^18^ The NHS England PCN coding was used to link the data sources. The total sum of patients registered with each network’s constituent practices was gathered from NHS Digital, which maintains a list of patients registered at each general practice. The March 2019 and 2022 releases were used to align with the study window.^19^

The income deprivation score was gathered from the Office for National Statistics English indices of deprivation 2019.^20^ The income deprivation domain score was used because it is a cardinal, continuous measure, representing the proportion of the population receiving benefits from the state owing to low income.

Each practice’s average was calculated using the distributions of their registered patients across Lower-layer Super Output Areas. The network average was calculated as the list-size weighted average of the constituent practices’ values.

#### Workforce data

Workforce data were gathered from NHS Digital. This included its Primary Care Workforce Quarterly Update,^21^ used for the ARRS and non- ARRS variables, while the other variables were gathered from its General Practice Workforce monthly updates.^22^ Data were gathered from March 2019 and March 2022, to define baseline and post-PCN workforce composition, respectively.

Workforce data are released at both network and practice level, with practice- level data requiring assignment to the network. Within the March 2022 workforce dataset, some practices were already assigned to a PCN. This information was used for most practice assignments. However, practices not assigned in 2022 were assigned according to the ODS classification.^18^ A number of practices remained unaligned, likely representing practices not enrolled in the PCN contract.

The workforce was disaggregated into full-time equivalent (FTE) total staff, total doctors, qualified GPs, doctors-in- training, nurses, direct patient care (DPC), administrative, ARRS, and non-ARRS. Within the DPC variable some healthcare workers were subsequently employed through the ARRS; making these workers both DPC and ARRS. To account for this issue, workers were class subsumed to still be ARRS in the March 2019 dataset. For example, a clinical pharmacist, an ARRS profession employed before the scheme, would be classed as ARRS and DPC in 2019 and 2022. Meanwhile, a healthcare assistant, which is a DPC staff type not included in ARRS, would be classified as non-ARRS and DPC throughout. The 2022 ARRS and non-ARRS variables, gathered from the quarterly release, included data from the ARRS and National Workforce Reporting Service (NWRS), used by general practices to report staffing. Meanwhile, the DPC and 2019 ARRS and non-ARRS variables are from the monthly release, which only uses the NWRS.

All workforce variables were standardised according to their network size, calculated as FTE per 10 000 patients.

### Deprivation rank

The deprivation rank of each PCN were calculated to measure deprivation-related inequality. PCNs were ranked from least to most deprived according to their average income deprivation score. The cumulative shares of the total population were then calculated. Each network was then assigned a fractional deprivation rank, the midpoint of their minimum and maximum cumulative share values.

For example, if the least deprived network had a combined list size representing 1% of the English population, it would have a fractional deprivation range of 0.00– 0.01. The fractional deprivation rank, the midpoint, would be 0.005. If the next least deprived network served 2% of the population, their deprivation range would be 0.01–0.03 with a fractional deprivation rank of 0.02. Instead, if the most deprived network served 5% of the population, its range would be 0.95–1.0 and rank 0.975.

### Slope index of inequality

The slope index of inequality (SII) is a standardised technique for calculating inequality.^23^^,^^24^ It represents the difference in the outcome variable between the least and most deprived networks. It is estimated using linear regression, with the sign of the regression coefficient indicating whether the variable is pro-rich or pro-poor. For example, a SII of 1 for the FTE GP variable would indicate 1 more FTE GP per 10 000 population in the most deprived network, compared with the least, while a SII of minus 1 would indicate 1 fewer.

The authors are interested in the change in the SII resulting from the PCN policy. Given workforce inequalities are significant and persistent in general practice,^2^^,^^3^ it is unlikely the PCN policy will have immediately corrected this association. Moreover, inequalities in workforce distribution were not a primary policy objective of the scheme. Rather, the aim was to increase the employment of other clinical staff in all general practices. However, ARRS recruitment may have improved or worsened such inequalities. As such it is the change in SII between 2019 and 2022, as opposed to the actual 2022 SII value, that is the study’s main focus.

### Adjustment for need

Most previous studies of geographical inequality in the distribution of primary care resources have adjusted for potential needs variables. There is little agreement, however, on how this is best done.

Given that the study is interested in the change in SII, as opposed to the actual SII, it was chosen to not control for potential needs factors, as it is unlikely that there would have been major shifts in the geographical distribution of underlying primary care needs between 2019 and 2022.

### Statistical analysis

Statistical analyses were conducted in Stata (version 17) MP4. Summary statistics were created for each workforce variable for the March 2019 and 2022 datasets. Change in the mean FTE workforce numbers per 10 000 patients were calculated. Linear regression with heteroskedasticity- robust standard errors was used to relate each of the workforce variables to the fractional deprivation rank. The estimated β coefficient is the SII for each time period. The data were pooled for 2019 and 2022 and included an indicator for 2022 and an interaction between this indicator and the fractional deprivation rank to estimate the change in the level of inequality. *T*-statistics and *P*-values were calculated for all analyses, weighted by population size.

## RESULTS

A final sample of 1255 networks was included. Mean network list size was 46 809 (95% CI = 45 739 to 47 878) in 2019 increasing to 48 913 (95% CI = 47 798 to 50 028) in 2022 (data not shown).

Summary statistics for the workforce variables in 2019 and 2022 are provided in Table 1. ARRS roles had the largest relative increase in size from 0.26 (95% CI = 0.24 to 0.28) to 3.08 (95% CI = 2.96 to 3.21). Meanwhile, qualified GPs (4.71 [95% CI = 4.62 to 4.79] to 4.50 [95% CI = 4.44 to 4.56]) and nurses (2.71 [95% CI = 2.64 to 2.78] to 2.65 [95 % CI = 2.59 to 2.71]) decreased.

**Table 1.** Summary statistics of the workforce variables by data collection point

**FTE staff per 10 000 patients**	**March 2019: mean (95% CI)**	**March 2022: mean (95% CI)**
Doctors	5.58 (5.48 to 5.67)	5.69 (5.61 to 5.78)
Qualified GPs	4.71 (4.62 to 4.79)	4.50 (4.44 to 4.56)
Doctors-in-training	0.87 (0.83 to 0.91)	1.19 (1.14 to 1.24)
Nurses	2.71 (2.64 to 2.78)	2.65 (2.59 to 2.71)
Direct patient care	1.89 (1.82 to 1.97)	2.45 (2.36 to 2.54)
Administrative	11.05 (10.82 to 11.29)	11.74 (11.56 to 11.91)
Total	21.23 (20.82 to 21.65)	22.53 (22.21 to 22.85)
ARRS	0.26 (0.24 to 0.28)	3.08 (2.96 to 3.21)
Non-ARRS	1.80 (1.29 to 2.31)	1.82 (1.74 to 1.89)

*ARRS = Additional Roles Reimbursement Scheme. FTE = full-time equivalent.*

Tables 2 and 3 show the results of the regression analysis. At the median deprivation rank, changes were observed in numbers of staff per 10 000 patients within many of the workforce categories. Small but significant increases in FTE total doctors, FTE doctors-in-training, FTE administrative, and FTE total staff were observed, while there were reductions in qualified GPs and nurses. An extra 2.828 (95% CI = 2.695 to 2.960) ARRS staff per 10 000 patients were observed in 2022 compared with 2019 when the staff were first introduced. Meanwhile, 0.555 (95% CI = 0.508 to 0.602) more FTE DPC were present in 2022, representing a 29.4% increase in this category over the 3 years. No significant change in total non-ARRS roles was observed.

**Table 2.** Slope index of inequality and median total numbers of doctors, administrative, nurses, direct patient care, and total staff; 2019 values and change in 2022 presented

**Category**	**FTE total doctors per 10 000 patients**		**FTE qualified GPs per 10 000 patients**		**FTE doctors-in-training per 10 000 patients**		**FTE administrative per 10 000 patients**		**FTE total staff per 10 000 patients**		**FTE nurses per 10 000 patients**		**FTE DPC per 10 000 patients**	
	**Coefficient (95% CI)**	***T* statistic**	**Coefficient (95% CI)**	***T* statistic**	**Coefficient (95% CI)**	***T* statistic**	**Coefficient (95% CI)**	***T* statistic**	**Coefficient (95% CI)**	***T* statistic**	**Coefficient (95% CI)**	***T* statistic**	**Coefficient (95% CI)**	***T* statistic**
Slope index of inequality in 2019	−0.802^a^ (−1.069 to −0.535)	−5.89	−0.750^a^ (−0.950 to −0.550)	−7.37	−0.051 (−0.178 to 0.075)	−0.79	0.960^a^ (0.459 to 1.461)	3.76	−0.626 (−1.551 to 0.300)	−1.33	0.117 (−0.071, 0.305)	1.22	−0.901^a^ (−1.146 to −0.657)	−7.23
Change in slope index of inequality	0.146 (−0.047 to 0.339)	1.48	−0.016 (−0.157 to 0.126)	−0.22	0.161^b^ (0.049 to 0.274)	2.83	−0.482^b^ (−0.841 to −0.122)	−2.63	−0.360 (−0.954 to 0.233)	−1.19	−0.013 (−0.126 to 0.101)	−0.22	−0.012 (−0.166 to 0.143)	−0.15
Value at median deprivation rank in 2019	5.576 (5.481 to 5.671)	115.11	4.706 (4.623 to 4.789)	111.32	0.871 (0.834 to 0.907)	46.49	11.05 (10.82 to 11.29)	91.81	21.23 (20.82 to 21.65)	100.61	2.707 (2.635 to 2.778)	74.05	1.894 (1.818 to 1.971)	48.62
Change at median deprivation rank	0.117^b^ (0.043 to 0.191)	3.10	−0.204^a^ (−0.258 to −0.151)	−7.47	0.321^a^ (0.286 to 0.356)	18.05	0.682^a^ (0.542 to 0.823)	9.55	1.298^b^ (1.052 to 1.545)	10.33	−0.056^b^ (−0.098 to −0.014)	−2.60	0.555^a^ (0.508 to 0.602)	23.41

*Coefficients from weighted least squares regression of levels of staff on fractional deprivation rank.*

a P<*0.001*.

b P<*0.01*.

*DPC = direct patient care. FTE = full-time equivalent.*

**Table 3.** Slope index of inequality and median total numbers of ARRS and Non-ARRS staff, 2019 values and change in 2022 presented

	**FTE ARRS per 10 000 patients**		**FTE non-ARRS per 10 000 patients**	
	**Coefficient (95% CI)**	***T* statistic**	**Coefficient (95% CI)**	***T* statistic**
Slope index of inequality in 2019	0.038 (−0.023 to 0.099)	1.22	−0.621^a^ (−1.240 to −0.002)	−1.97
Change in Slope index of inequality	0.221 (−0.095 to 0.538)	1.37	−0.332 (−0.899 to 0.235)	−1.15
Value at median deprivation rank in 2019	0.257 (0.237 to 0.277)	25.16	1.801 (1.289 to 2.312)	6.90
Change at median deprivation rank	2.828^b^ (2.695 to 2.960)	41.90	0.018 (−0.470 to 0.506)	0.07

*Coefficients from weighted least squares regression of levels of staff on fractional deprivation rank.*

a P<*0.05.*

b P<*0.001.*

*ARRS = Additional Roles Reimbursement Scheme. FTE = full-time equivalent.*

Deprivation-related inequalities were observed in staff distribution per 10 000 patients at baseline. In 2019, 0.802 (95% CI = 1.069 to 0.535) fewer FTE total doctors, 0.901 (95% CI = 1.146 to 0.657) fewer FTE DPC, and 0.621 (95% CI = 1.240 to 0.002) fewer FTE non-ARRS roles per 10 000 patients were employed in the most deprived network compared with the least (pro-rich). Meanwhile, 0.960 (95% CI = 0.459 to 1.461) more FTE administrative staff were employed in the most deprived network compared with the least. There was a slight pro-poor distribution in nurses and ARRS roles at baseline, but this did not reach statistical significance (Tables 2 and 3).

Over the study period, changes in this unequal distribution were observed, part of which is likely to be owing to the ARRS. FTE administrative staff became less pro- poor, where 0.482 (95% CI = 0.841 to 0.122; *P*<0.01) fewer FTE administrative staff per 10 000 patients were employed in the most deprived network relative to the least, when comparing with the 2019 distribution (Table 2). FTE total doctors and FTE ARRS became more pro-poor over the study period, with 0.146 (95% CI = −0.047 to 0.339) and 0.221 (95% CI = −0.095 to 0.538) more FTE total doctors and FTE ARRS, respectively, relative to their 2019 distribution (Tables 2 and 3). However, when differentiating between subtype of doctor, the distribution of qualified GPs was observed to become more pro-rich (−0.016 [95% CI = −0.157 to 0.126]) and doctors- in-training was observed to become pro-poor (0.161 [95% CI = 0.049 to 0.274; *P*<0.01]) (Table 2). FTE nurses, FTE DPC, FTE total staff, and FTE non- ARRS became more pro-rich over the study period (Tables 2 and 3). Only doctors- in- training changed from pro- rich to pro-poor (Table 2). For example, FTE GPs, which were significantly pro-rich in 2019, became more pro-poor over the study period, but remained pro-rich overall.

**Figure 1.**
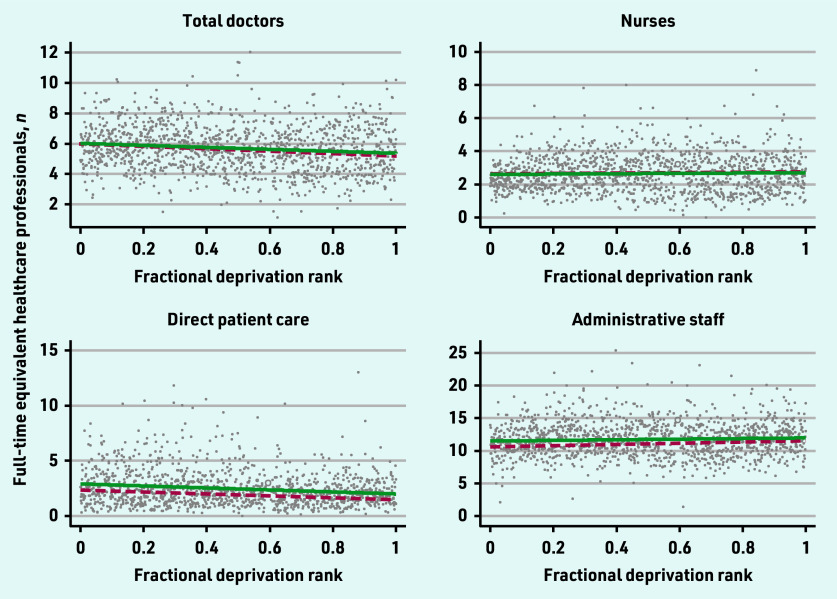
*Scatterplot of FTE total doctors, nurses, direct patient care, and administrative staff per 10 000 patients by the primary care network fractional deprivation rank. The line indicates the slope index of inequality, with the red dashed line detailing the March 2019 slope and the green line the March 2022 slope.*

**Figure 2.**
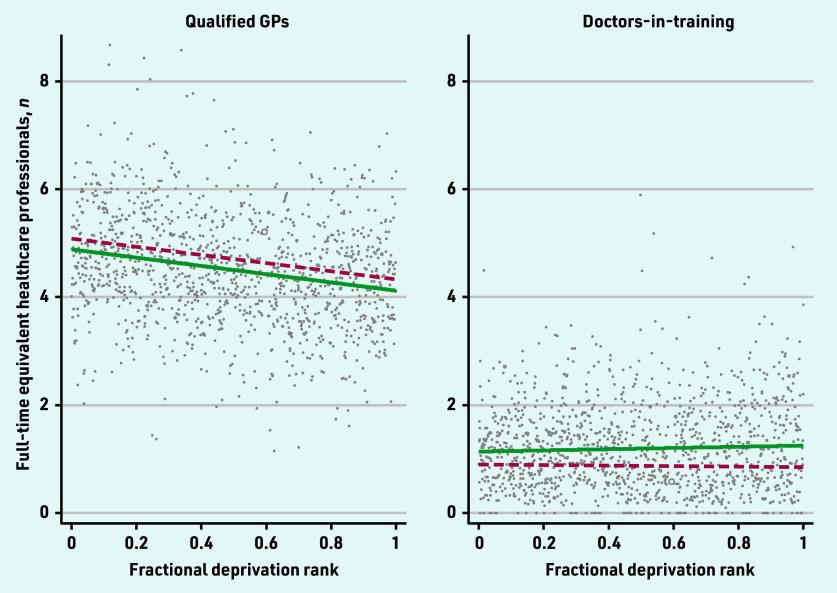
*Scatterplot of full-time equivalent qualified GPs and doctors- in-training per 10 000 patients by the primary care network fractional deprivation rank. The line indicates the slope index of inequality, with the red dashed line detailing the March 2019 slope and the green line the March 2022 slope.*

**Figure 3.**
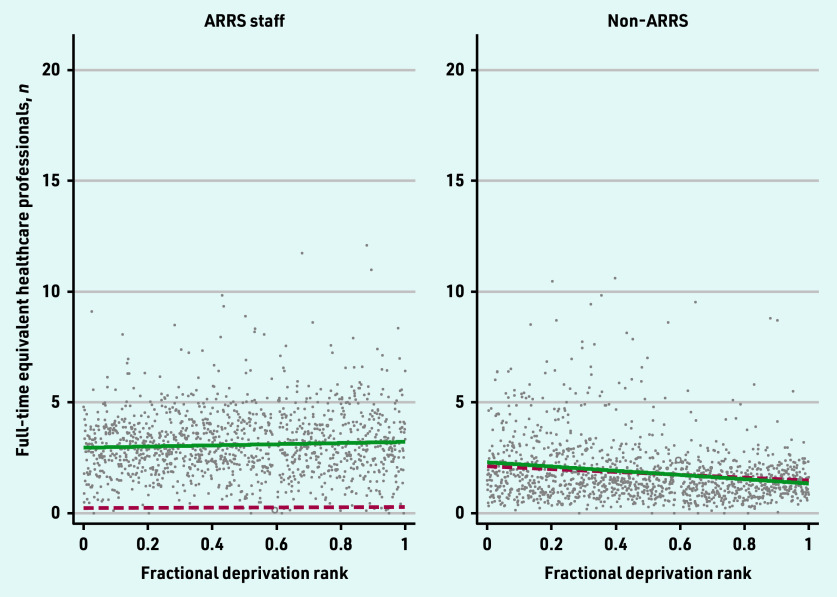
*Scatterplot of full-time equivalent ARRS and non-ARRS staff per 10 000 patients by the primary care network fractional deprivation rank. The line indicates the slope index of inequality, with the red dashed line detailing the March 2019 slope and the green line the March 2022 slope. ARRS = Additional Roles Reimbursement Scheme.*

The 2019 and 2022 SII for the workforce categories are presented in Figures 1–3.

## DISCUSSION

### Summary

The ARRS saw an increase in associated staff employed in general practice, alongside all pre-existing staff types, except qualified GPs and nurses, which reduced. A statistically significant pro- rich change (*P*<0.01) in the distribution of administrative staff was observed since the ARRS, alongside trends towards pro- rich changes in qualified GPs, nurses, DPC, total staff, and non-ARRS staff, which did not reach statistical significance. Pro- poor changes in total doctors, doctors- in- training, and ARRS staff were observed, with only the change in doctors-in-training reaching statistical significance (*P*<0.01).

Pre-existing inequalities in workforce distribution was congruent with previous studies,^3^^,^^6^ with only doctors-in-training changing from pro-rich to pro-poor.

### Strengths and limitations

This study included widely used datasets covering all PCNs enabling the analysis of the impact the ARRS had on inequality of the general practice workforce.

General practices will enter and exit the market at irregular intervals, meaning the set of practices comprising PCNs will be different in 2019 and 2022. Staff working at practices in 2022 will have come in part from practices in 2019. Further, the monthly DPC data does not include all ARRS data in the quarterly release, meaning the sum of ARRS is observed and non-ARRS is greater than total DPC. Similarly, the analysis relies on the quality of NHS workforce data, which is sometimes criticised. This is the best data available to answer the research question and quality is gradually improving.

March 2019 was chosen as it is the closest datapoint to the ARRS implementation. It is felt that this has provided adequate time for the staff employment, but the clinical outcomes of these changes may take more time to mature. Similarly, over this time period the COVID-19 pandemic occurred, which is a potential confounding factor. Other policies may be influencing factors; for example, the targeted enhanced recruitment scheme incentivises doctors- in-training to work in deprived areas independent of the ARRS.^25^

SII is a robust methodology, widely used in studies of inequality.^23^^,^^24^ However, as the mean of the outcome increases, the SII will increase, even if relative inequality remains the same. Only relatively small changes in mean workforce numbers were observed, except for ARRS and DPC staff, which will be relatively overestimated. This will not impact the pro-rich or pro-poor relationship.

### Comparison with existing literature

Workforce inequalities in general practice are longstanding^2^^–^4 and some changes were found associated with the ARRS. Some staff groups follow a pro-poor distribution (nurses, administrative, and ARRS) and others pro-rich (total doctors, qualified GPs, DPC, total staff, and non-ARRS). Slight variations of specific staff groups are noted between studies, owing to the different time periods used. For example, Nussbaum *et al* found a pro-rich distribution for nurses between 2015 and 2020 but identified that this was transitioning to pro-poor.^5^

Recent evidence has demonstrated how interventions in primary care that are not designed around inequalities can unintentionally exacerbate them.^8^ The ARRS was not specifically designed as a mechanism for reducing inequalities in staff distribution, but it remains important to understand its impact. This is particularly true given concerns about the inequity in the PCN funding formulae and the potential issues for ARRS staff recruitment in deprived areas.^15^

The findings of the present study suggest that the ARRS has not significantly exacerbated existing deprivation-related geographical inequities in the clinical workforce, with a trend towards ARRS staff marginally favouring more deprived practices. However, there has been some change in administrative staff employment. Historically, this favoured more deprived practices, but the extent of this has diminished alongside the ARRS. This may reflect differential employment of PCN- associated administrative staff in less deprived practices.

### Implications for research and practice

The ARRS is associated with a reduction in the pro-poor distribution of administrative staff, with a possible trend towards the distribution of qualified GPs, nurses, DPC, total staff, and non-ARRS staff becoming more pro-rich. This needs to be observed over time, as it may indicate potential issues with ARRS making other types of recruitment more difficult in deprived areas. Fortunately, the ARRS staff themselves are distributed pro-poor and total doctors distributed less pro-rich, although qualified GPs have become more so.

The reduction of the pro-poor distribution of administrative staff is concerning as they often facilitate access to the NHS, meaning they play an important role in addressing unmet needs in deprived communities.^26^^–^28

Existing research has suggested opportunities to encourage pro-poor changes, particularly with GPs, DPC, and non-ARRS staff.^5^^,^^29^ This could be achieved by increasing the weighting of funding, directly incentivising posts, or improving workforce compensation in deprived communities.^5^^,^^29^ Research has found heterogeneity in PCNs, with some covering relatively homogeneous deprived or affluent populations, while others have varying levels of deprivation.^30^

PCNs, which cover varied populations, could actively deploy staff to mitigate existing inequalities, an issue that needs further research. There may be other approaches beyond increasing staff numbers that could improve patient access, such as demand management strategies or the use of technology.

Finally, increasing the number of trainees recruited from deprived communities, as well as improving the training in these communities, may improve recruitment and retention.^31^^,^^32^

## Data

Data available on request to lead author.
